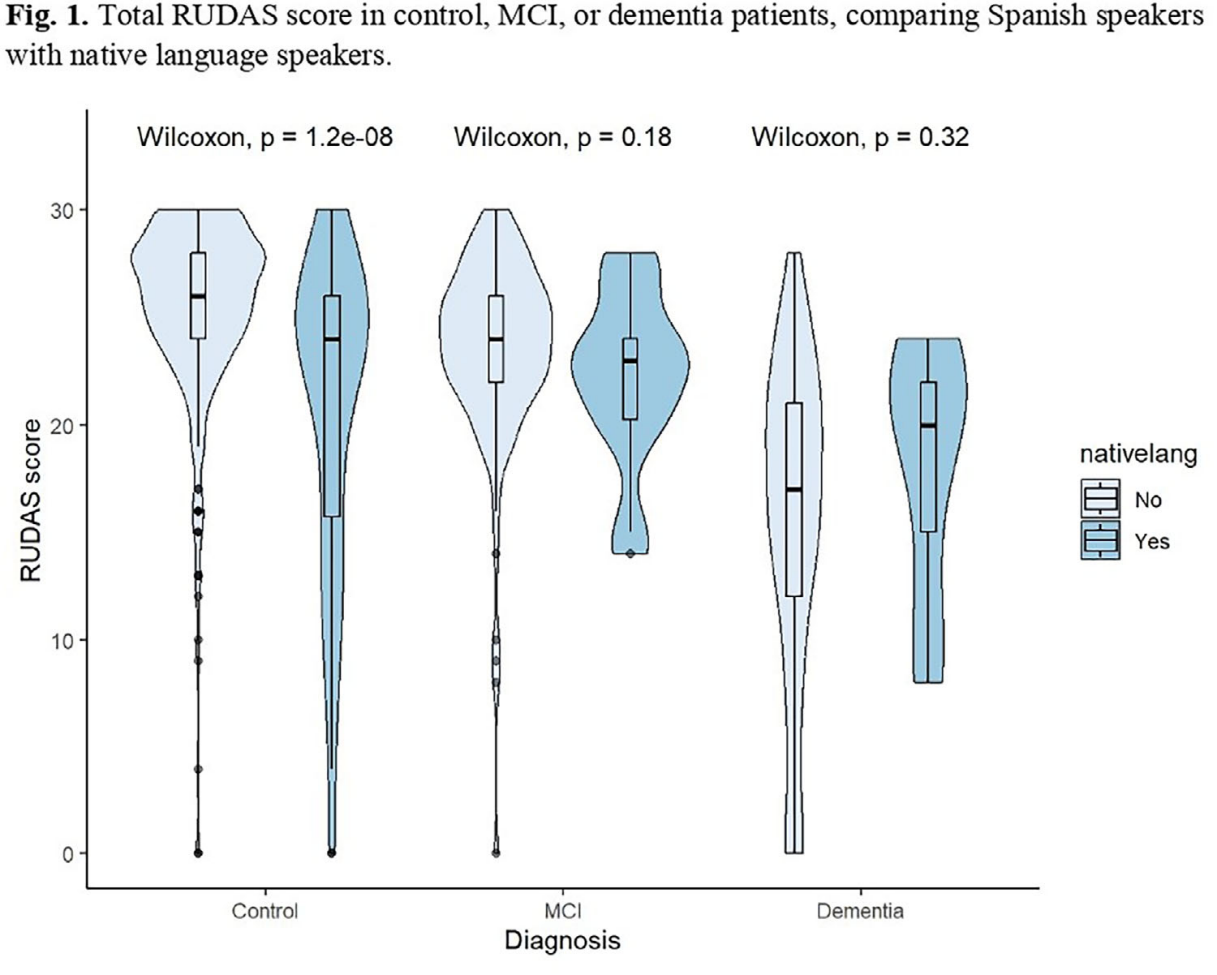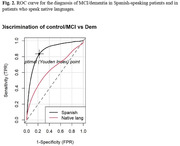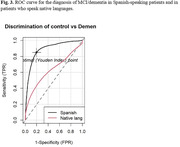# Assessing Cognitive Impairment in Quechua and Aymara Patients: A Critical Examination of the Rowland Universal Dementia Assessment Scale’s Discriminative Capacity

**DOI:** 10.1002/alz.093273

**Published:** 2025-01-03

**Authors:** Marco Malaga, Diego Bustamante‐Paytan, Arturo Jhonny Ruiz‐Yaringaño, Belen Custodio, Marcio F. Soto‐Añari, Maria Fernanda Ore‐Gomez, Juan Carlos Duran Quiroz, María Isabel Cusicanqui, Rosa Montesinos, Nilton Custodio, Giuseppe Tosto

**Affiliations:** ^1^ Cognitive Impairment Diagnosis and Dementia Prevention Unit, Instituto Peruano de Neurociencias, Lima, Perú, Lima, Lima Peru; ^2^ Cognitive Impairment Diagnosis and Dementia Prevention Unit, Peruvian Institute of Neurosciences, Lima, Lima Peru; ^3^ Sociedad Científica de San Fernando, Lima, Lima Peru; ^4^ Unit Cognitive Impairment and Dementia Prevention, Peruvian Institute of Neurosciences, Lima, Peru, Lima, Lima Peru; ^5^ Universidad Católica San Pablo, Arequipa Peru; ^6^ Instituto Peruano de Neurociencia, Lima Peru; ^7^ Universidad Mayor De San Andres, La Paz Bolivia (Plurinational State of); ^8^ Centro Neurológico Mente Activa, La Paz Bolivia (Plurinational State of); ^9^ Taub Institute for Research on Alzheimer’s Disease and the Aging Brain, Vagelos College of Physicians and Surgeons, Columbia University, New York, NY USA

## Abstract

**Background:**

The global aging population raises concerns about increased neurodegenerative diseases, particularly in low‐ and middle‐income countries like Latin America and the Caribbean. However, the situation among the indigenous inhabitants remains unknown due to various barriers, including cultural diversity, lack of studies, low awareness, language barriers, and limited healthcare access. Brief cognitive tests like the Rowland Universal Dementia Assessment Scale (RUDAS) show promise in overcoming these challenges.

**Method:**

A secondary analysis was conducted on a substantial patient cohort derived from the GAPP study (NIA grant #alz093273AG069118), categorizing participants based on whether their native language was Spanish or Quechua/Aymara. Descriptive analysis of variables was performed, with the Wilcoxon test applied to compare total RUDAS scores among distinct patient groups, including controls, individuals with Mild Cognitive Impairment (MCI), and those with dementia. Comparisons were specifically made between Spanish speakers and native language speakers. Additionally, the ROC (Receiver Operating Characteristic) curve was employed to evaluate the discriminative capacity of RUDAS in distinguishing between controls and dementia, as well as between controls/MCI and dementia cases within native and Spanish‐speaking populations.

**Result:**

A total of 405 controls, 126 individuals with Mild Cognitive Impairment (MCI), and 133 participants with dementia were included in the analysis. Among them, 91 patients were native language speakers, with a median age of 74 (range 54 ‐ 92). The majority were female (69.2%), and 65.9% were Aymara speakers. Native participants reported an average education level of 3.1 years (SD 4.4). The RUDAS mean score was lower in the native speaker group compared to Spanish‐speaking participants (21 vs. 23). Additionally, the analysis revealed significantly lower scores in controls within the native speaker group (Fig. 1). Diagnostic performance for native speakers was poor when comparing control/MCI (Fig. 2) vs. dementia and control vs. dementia (Fig. 3).

**Conclusion:**

The use of the RUDAS in native language speakers and its ability to distinguish between Mild Cognitive Impairment (MCI) and dementia raise some concerns. To improve assessments for various communities in Latin America and the Caribbean, there is a need for culturally adapted brief cognitive tests.